# Hypertension: Pathophysiological Aspects, Psychosocial Stress and Food Preference

**DOI:** 10.5935/abc.20190202

**Published:** 2019-09

**Authors:** Heno Ferreira Lopes

**Affiliations:** Instituto do Coração - Hospital das Clínicas da Faculdade de Medicina da Universidade de São Paulo, São Paulo, SP - Brazil

**Keywords:** Hypertension, Obesity, Diabetes Mellitus, Dyslipidemias, Sedentary Behavior, Oxidative Stress, Healthy Diet, Indicators of Morbidity and Mortality

Arterial hypertension is highly prevalent in developed and developing countries. Together with high blood glucose levels, hyperlipidemia, overweight and obesity, it is considered a consequence of behavioral risk factors such as physical inactivity, tobacco use, harmful alcohol use and inadequate diets.^1^ The cause of arterial hypertension in most of the cases (over 90%) is unknown. The activation of the sympathetic nervous system, the renin-angiotensin-aldosterone system and an abnormal pressure-natriuresis curve play an important role in the pathophysiology of hypertension.^2^ Oxidative stress has also been identified as an intermediate phenotype in the development of arterial hypertension.^3^ Experimental and epidemiological studies point to psychosocial stress as a possible trigger that causes the autonomic imbalance (increased sympathetic activity) in hypertensive patients.^4^ This autonomic imbalance can be observed even before the arterial hypertension onset in children born to hypertensive parents.^5^ In addition to psychosocial stress, an unhealthy diet contributes to the development of arterial hypertension and higher cardiovascular morbidity/mortality.^6^

If, on the one hand, diet plays an important role in the pathophysiology of arterial hypertension, on the other hand, adopting a healthy diet can result in better blood pressure control. The DASH (Dietary Approach to Stop Hypertension) diet, mentioned in worldwide guidelines, was evaluated by Appel et al.^7^ and it was the first scientifically tested diet to result in a significant blood pressure reduction in hypertensive patients. The DASH diet consists of easily accessible foods such as vegetables, fruits, nuts, lean meat, and low-fat milk and dairy products. In a study of obese hypertensive patients,^8^ we tried to elucidate the possible mechanisms involved in blood pressure reduction after the consumption of the standard DASH diet. In this study we demonstrated that the consumption of a standard DASH diet results in improved antioxidant capacity, especially in obese hypertensive patients. Since oxidative stress plays a role in the pathophysiology of arterial hypertension, this is one of the possible mechanisms for reducing blood pressure in those individuals who consume the DASH diet foods in adequate proportion.

Although there is a previously tested diet with a positive impact on blood pressure reduction, as in the case of the DASH diet, there is a tendency among humans to preferentially consume some types of food. Previous studies, mainly experimental ones, have evaluated the association of stress exposure and emotional state with preference for some specific foods.^9^

In the article by Ulrich-Lae et al.,^9^ they describe the association of eating high-fat and sweet foods with stress improvement in animals. As the stressed animals and human beings prefer more caloric foods (carbohydrates and fats), the tendency is to develop obesity. Obesity is known to be directly related to arterial hypertension.^10^

In this issue of the Brazilian Archives of Cardiology, Dalmazo et al.^11^ demonstrated the association of stress levels with a higher consumption of high-fat foods in patients with arterial hypertension. The findings of this study indicate the importance of a multidisciplinary approach in hypertensive patients, especially those with high levels of psychosocial stress.
